# Performance and Transcriptional Response of the Green Peach Aphid *Myzus persicae* to the Restriction of Dietary Amino Acids

**DOI:** 10.3389/fphys.2020.00487

**Published:** 2020-05-25

**Authors:** Jun Wu, Hao Lan, Zhan-Feng Zhang, He-He Cao, Tong-Xian Liu

**Affiliations:** State Key Laboratory of Crop Stress Biology for Arid Areas and Key Laboratory of Integrated Pest Management on Crops in Northwestern Loess Plateau, Ministry of Agriculture, Northwest A&F University, Yangling, China

**Keywords:** amino acid restriction, RNA-seq, transcriptional plasticity, amino acid metabolism, glycolysis, green peach aphid

## Abstract

Free amino acids in the phloem sap are the dominant nitrogen source for aphids, but their availability is usually poor. Although some studies have explored the effect of dietary amino acid restriction on aphid performance, little is known about the molecular basis of these effects. Here, we examined the performance and transcriptome of the green peach aphid, *Myzus persicae*, fed a standard diet (Control diet) or a diet containing 50% of the total amino acids of the Control diet (Half diet). Aphid weight and fecundity were significantly reduced in the Half diet group. Transcriptomic analysis showed that a total of 1460 genes were differentially expressed between the groups were fed on the two diets, which many of them were associated with nutrient and energy metabolism. When feeding on the Half diet, aphids upregulated genes associated with the amino acid biosynthetic pathway (predominantly amino acid biosynthesis genes and some amino acid transporter genes) as well as the cysteine and serine protease genes. Furthermore, these aphids displayed increased expression of genes associated with glycolysis, which could generate intermediates for *de novo* amino acid biosynthesis. Consistent with this, elevated glucose levels were observed in aphids in the Half diet group. Additionally, the expression levels of several genes associated with hormonal signaling pathway were altered. Several genes related to juvenile hormone and insulin-like peptide (ILP) signaling were downregulated, including *Krüppel homolog 1* (*Kr-h1*) and *insulin-like peptide 5* (*Ilp5*), respectively. In contrast, several genes related to ecdysone signaling were upregulated including *broad-complex core protein* (*Br-c*) and *shade* (*Shd*). Despite their poor performances, *M. persicae* adapted to dietary restriction of amino acids, through upregulation of genes involved in amino acid biosynthesis, glycolysis, and protein degradation, as well as by altering the expression level of genes involved in hormone signaling pathways.

## Introduction

The green peach aphid *Myzus persicae*, a notorious phloem-feeding hemipteran pest, colonizes more than 400 different plant species from over 50 families (Blackman and Eastop, 2000), and is capable of transmitting more than 100 plant viruses. This causes enormous reductions in crop yield, both through direct feeding and by transmission of viruses (Beemster and De Bokx, 1987). Availability of nitrogen as a nutrient source in plants generally limits the performance of phytophagous insects (Douglas, 2006). Amino acids in plant phloem sap are the dominant dietary nitrogen source for phloem-feeding hemipterans (Ziegler, 1975; Rahbé et al., 1990; Douglas, 1993). As the sole food source for phloem-feeding hemipterans (Douglas, 2006), phloem sap is generally considered a nutritionally unbalanced sucrose-rich diet, but deficient in amino acids (Karley et al., 2002; Douglas, 2006; Dinant et al., 2010). In addition, the amino acid contents of phloem sap differ among different host plant species, and even among different development stages, tissues, or growth conditions within a single host plant (Hayashi and Chino, 1990; Corbesier et al., 2001; Douglas, 2003, 2006; Wilkinson and Douglas, 2003; Gholami, 2004). Given that amino acid availability significantly affects growth and reproduction of aphids (Simpson et al., 1995; Ponder et al., 2000), examination of the mechanisms underpinning adaptation to dietary amino acid restriction are necessary. Previous studies mainly focused on the impact of amino acid restriction on aphid performance, using chemically defined artificial diets or host plants (Simpson et al., 1995; Ponder et al., 2000; Hunt et al., 2009). However, the molecular mechanisms underlying the adaptation to dietary restriction of amino acids largely remain unknown.

Aphid performance is significantly affected by the total intake of dietary amino acids. Although most studies show that restriction of dietary amino acids reduces aphid performance (Simpson et al., 1995; Ponder et al., 2000; Hunt et al., 2009), specific performance responses vary between different aphid species. Previous studies of two aphid species, the pea aphid (*Acyrthosiphon pisum*) and the bird cherry-oat aphid (*Rhopalosiphum padi*), reared on a chemically defined artificial diet or a host plant, respectively, showed that dietary amino acid restriction reduced survival rate, growth rate, and fecundity (Simpson et al., 1995; Ponder et al., 2000). However, previous studies of *M. persicae*, using *Arabidopsis thaliana aap6* mutant plants with reduced total phloem sap amino acids compared to wildtype plants, revealed a minor effect on aphid reproduction (Hunt et al., 2009). Furthermore, most studies show that the free amino acid pool in the body of phloem-feeding hemipterans changes according to dietary amino acid composition (Liadouze et al., 1995; Crafts-Brandner, 2002; Moran et al., 2005). Stable isotope studies of aphid amino acid metabolism revealed that free amino acids within the aphid body are mostly the product of dietary amino acid catabolism and subsequent re-synthesis (Haribal and Jander, 2015). These studies provide clear evidence that aphids have the ability to adjust amino acid metabolism according to dietary amino acid availability. Therefore, we inferred that metabolic networks of amino acids may play an important role in the adaptation to dietary amino acid availability.

In order to determine potential molecular mechanism underlying the adaptation to dietary restriction of amino acids, we examined the performance and transcriptional responses of *M. persicae* fed chemically defined artificial diets with two different dietary amino acid profiles. We also measured amino acid and sugar content in *M. persicae* by liquid chromatography–mass spectrometry (LC-MS). Specifically, the present study aimed to determine how the restriction of dietary amino acids affects: (1) the performance of *M. persicae*; (2) the free amino acid pool and expression of metabolic genes; and (3) the expression of genes in hormone signaling pathways. The findings obtained here provide insights into the molecular mechanisms of adaptation to dietary restriction of amino acids in *M. persicae* and other phloem-feeding pests.

## Materials and Methods

### Aphids and Artificial Diet

Laboratory colonies of *M. persicae* were collected from cabbage (*Brassica oleracea*; c.v. “Qingan 70”) in the greenhouse of Northwest A&F University (Yangling, Shaanxi, China) at temperatures ranging from 18 to 28°C, and under natural conditions in 2016. The apterous parthenogenetic aphids were maintained on cabbage under a controlled environment room at 24 ± 2°C temperature with a 16/8 h photoperiod as laboratory colony. The standard diet (Control diet) used was previously described (Febvay et al., 1988).

To detect physiological changes and the molecular mechanisms necessary for adaptation to the dietary restriction of amino acids in *M. persicae*, we manipulated their diet. We provided either a standard artificial diet (Control) or a restricted (Half) diet. Control and Half diets were both prepared with stock solutions of 2 × (amino acids), 10 × (vitamins), and 10 × (trace metals), and stored at −80°C if not used at once. Diet components are outlined in Supplementary Table S1. The levels of sucrose, minerals, and vitamins of Half diet were identical with Control diet, but the concentrations of all amino acids were reduced by 50%. No other changes were made to the diets of the two groups.

### Artificial Diet Feeding Trial Bioassays

Apterous adults were removed from the host plant with a small brush and then placed onto an intact cabbage for 24 h for producing nymphs. After this period, newborn nymphs were transferred to one of the artificial diets. After transferred to diet for 6 days, aphids (fourth instar nymphs) feeding on both Control and Half diets were collected for weight measurement on a micro-balance (Sartorius MSA 3.6P-000-DM, Göttingen, Germany). Each diet group comprised 6 subgroups (∼15 aphids per subgroup). The mean weight of each subgroup was used to data analysis and data were analyzed using Student’s *t*-test. To test the pre-reproduction period, the number and the time point of aphids begun producing nymphs was recorded every 6 h after transferred to diet for 7 days (the time point of transition to adulthood in aphids reared on both Control and Half diet groups). Half and Control diet groups were composed of totally 60 and 55 individual aphids, respectively. The data were analyzed using Student’s *t*-test. To test the fecundity and lifespan of aphids feeding on Control and Half diets, the number of adult and offspring were counted every 2 days and the diets were replaced every 2 days until all aphids were dead. To the fecundity test, each diet group comprised six subgroups (∼15 aphids per subgroup). The average number of offspring per adult per day of each subgroup was used to data analysis and data were analyzed using Student’s *t*-test. To the lifespan test, Half and Control diet groups were composed of totally 88 and 91 individual aphids, respectively. The number of dead aphids was recorded every 2 days. Data were analyzed using Log Rank Test. All experiments were carried out at 24 ± 1°C, 70% ± 5% relative humidity, and 16-h light period in a climate chamber.

### Amino Acids, Sugars, and Total Protein Analysis

To detect the effect of dietary amino acid restriction on the metabolite profiles of aphids, we measured the abundance of amino acids, sugars and total protein in aphids feeding on either Control or Half diets for 6 days. The amino acids and sugars in aphids were extracted with 50 mM HCl by grinding with a glass pestle and mortar on ice. Amino acids and sugars extracted from the whole body of aphids were analyzed as previously described (Cao et al., 2016). Each diet group comprised eight subgroups. One subgroup contained 10 mg fresh weight Day 6 aphids (fourth instar nymphs) were fed on Half or Control diet. The mean concentration of amino acids and sugars of eight subgroups was used to data analysis and data were analyzed using Student’s *t*-test. Total protein contents in aphids feeding on Control and Half diets for 6 days were measured using a BCA protein assay kit (Sangon Biotech Co., Ltd., Shanghai, China). Each diet group comprised seven subgroups. One subgroup contained 5 mg fresh weight Day 6 aphids were fed on Half or Control diet. The mean total protein concentration of seven subgroups was used to data analysis and data were analyzed using Student’s *t*-test.

### RNA Sequencing

Sixty *M. persicae* nymphs that feeding on Control and Half diets for 6 days (fourth instar nymphs), respectively, were collected, immediately frozen in liquid nitrogen, and stored at −80°C for RNA extraction. Three independent biological replicates were performed for RNA sequencing analysis. Total RNA was extracted from the whole body of aphids using RNAiso Plus (Takara Biotechnology Co., Ltd., Dalian, Liaoning, China) following the manufacturer’s instructions. Samples were then quantified by Qubit2.0^®^ Fluorometer (Life Technologies, New York, NY, United States) and qualified by Agilent 2100 (AgilentTechnologies, Palo Alto, CA, United States). High quality RNA was used for cDNA synthesis and Illumina library generation that were completed at the Novogene Bioinformatics Technology Co., Ltd. (Beijing, China). The average proportion of clean reads in each sample was 97.99–98.18%. The filtered clean reads from each sample were aligned to the reference *M. persicae* genome by HISAT software (version 2.0.4) with total mapping rate 95.33–95.79% (Kim et al., 2015).

### Functional Annotation and Enriched Pathways of Differentially Expressed Genes (DEGs)

We used HTSeq (0.6.1) to count the reads numbers mapped to each gene. Then, the expected number of fragments per kilobase of transcript sequence per millions base pairs sequenced (FPKM) calculated based on the length of the gene and reads count mapped to this gene, and the gene expression levels were estimated as described previously (Trapnell et al., 2010). Differential expression analysis of two diet groups was performed using the DESeq R package (1.10.1) (Anders and Huber, 2010). Genes with an adjusted *p*-value < 0.05 found by DESeq were assigned as differentially expressed.

To find out differentially expressed genes (DEGs) involved in adaptation to an amino acid-restricted diet in *M. persicae*, we functionally annotated and identified putative biological pathways of DEGs using the gene ontology (GO) and the Kyoto Encyclopedia of Genes and Genomes (KEGG) enrichment analysis. GO enrichment analysis of DEGs was implemented by GOseq R package (2.12) (Young et al., 2010) and the statistical enrichment of DEGs in KEGG pathways were analyzed by the KEGG Orthology-Based Annotation System (KOBAS) software (2.0) (Mao et al., 2005). Heatmaps of DEGs showed in Results section were constructed with binary log of fold-change of Half/Control for each gene in TBtools software (Chen et al., 2018).

### cDNA Synthesis and Real-Time Quantitative PCR (RT-qPCR) Analysis

Single-stranded cDNA was synthesized from 1 μg total RNA using the PrimeScript^TM^ RT reagent Kit with gDNA Eraser (Perfect Real Time, TaKaRa, Dalian, Liaoning, China). To validate transcriptome data, we randomly selected 15 DEGs and quantified the expression level of these genes using RT-qPCR method.

The ribosomal protein L7 (RPL7) was selected as the reference gene for RT-qPCR as described previously (Tzin et al., 2015). The total reaction volume (20 μL) comprised 10 μL 2 × SYBR Premix Ex Taq II (Tli RNaseH Plus, Takara), 1 μL primer mix (150 nM final concentration of each primer), and 9 μL diluted cDNA. RT-qPCR was performed on a LightCycler^®^ 480 System (Roche, Basel, Switzerland) with cycling conditions comprising: 5 min at 95°C, 40 cycles of 95°C for 10 s, 60°C for 30 s. The results of the RT-qPCR were normalized to the expression level of RPL7 and calculated by the 2^–ΔΔ^ ^Ct^ method (Livak and Schmittgen, 2001).

### Statistical Analysis

Statistical analyses were performed with SPSS software, version 20 (Armonk, NY, United States: IBM Corp.). Two group comparisons were analyzed by the Student’s *t*-test. We considered *p* < 0.05 as statistically significant.

## Results

### Aphid Performance on Standard and Amino Acid-Restricted Diets

**FIGURE 1:**
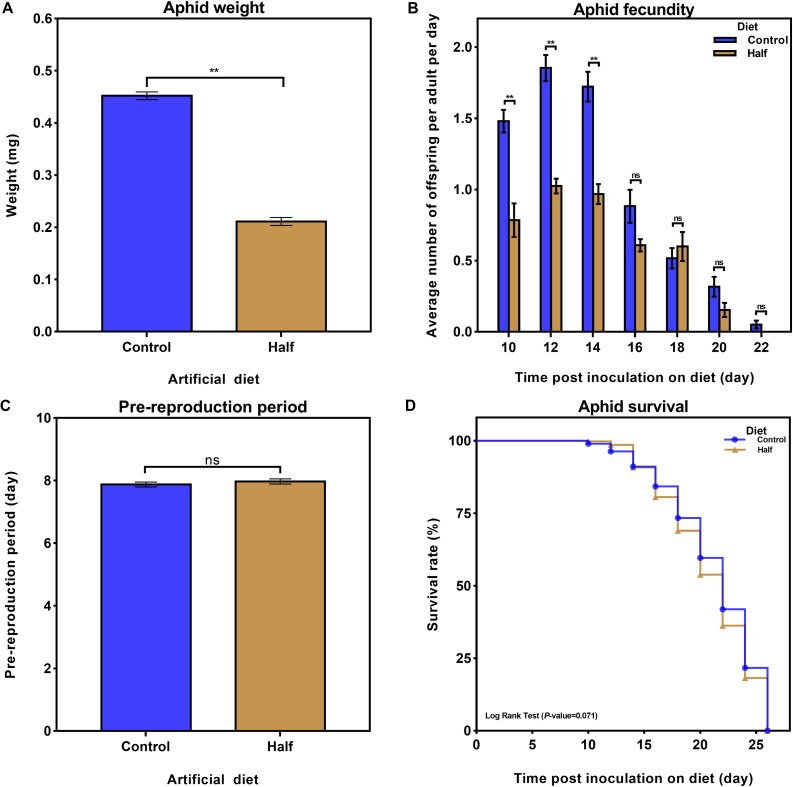
Effects of the reduced dietary amino acid level diet on Myzus persicae performance. **(A)** Effects of the amino acid-restricted diet (Half diet, yellow) and normal diet (Control diet, blue) on aphid weight six days after transfer to artificial diets. Results are reported as mean (±SE) of six subgroups (∼15 aphids per subgroup) per diet group. Data were analyzed using Student’s *t*-test (**p < 0.01). **(B)** Average number of nymphs per aphid per day fed the Half and Control diets. Results are reported as mean (±SE) of six subgroups (∼15 aphids per subgroup) per diet group. Data were analyzed using Student’s *t*-test (ns, not significant; **p < 0.01). **(C)** Effect of the Half and Control diets on aphid pre-reproduction period. Half and Control diet groups were composed of 60 and 55 aphids, respectively. Data were analyzed using Student’s *t*-test (ns, not significant). **(D)** Effects of the Half and Control diets on aphid survival. Half and Control diet groups were composed of 88 and 91 aphids, respectively. Data were analyzed using Log Rank Test.

To detect the aphid performance response to dietary restriction of amino acids, we recorded different parameter as metrics of *M. persicae* performance: weight, fecundity, and pre-reproduction period, as well as lifespan. The restriction of dietary amino acids decreased the weight of aphids (Figure 1A, *p* < 0.01, Student’s *t*-test), and their fecundity of aphids (Figure 1B, *p* < 0.01, Student’s *t*-test) but did not affect the pre-reproduction period (Figure 1C, *p* = 0.394, Student’s *t*-test) and lifespan (Figure 1D, *p* = 0.071, Log Rank Test).

### Amino Acids, Sugars, and Total Protein Content in Aphids

**FIGURE 2:**
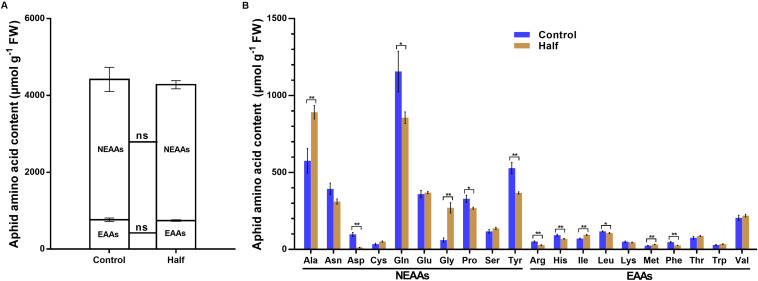
Amino acid content of *Myzus persicae* fed the Half and Control diets. **(A)** Total essential amino acids (EAAs) and total non-essential amino acids (NEAAs) contents of aphids fed the Half and Control diets. Data were analyzed using Student’s *t*-test (ns, not significant). **(B)** The amino acids content of aphids fed the Half and Control diets. Results are reported as mean (±SE) of eight subgroups (10 mg aphids per subgroup) per diet group. Data were analyzed using Student’s *t*-test (*P < 0.05; **P < 0.01).

**FIGURE 3:**
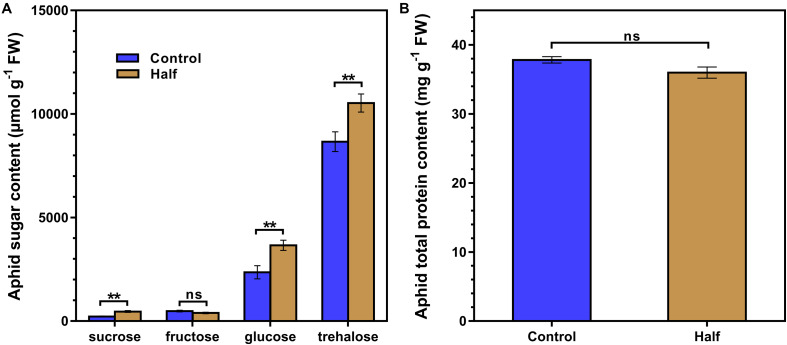
Sugar and total protein contents of *Myzus persicae* fed the Half and Control diets. **(A)** The sugar content of aphids fed the Half and Control diets. Results are reported as means (±SE) of eight subgroups (10 mg aphids in per subgroup) per diet group. Data were analyzed using Student’s *t*-test (ns, not significant; **P < 0.01). **(B)** Total protein content of aphids fed the Half and Control diets. Results are reported as means (±SE) of seven subgroups (5 mg aphids per subgroup) per diet group. Data were analyzed using Student’s *t*-test (ns, not significant).

The restriction of dietary amino acids did not affect the total amount of non-essential amino acids (NEAAs) and essential amino acids (EAAs) (Figure 2A) but significantly affected the amino acid profiles in aphids (Figure 2B). All 20 amino acids were detected in aphids fed the Half and Control diets (Figure 2B). The concentrations of four NEAAs (aspartate, glutamine, proline and tyrosine) significantly decreased in aphids fed the amino acid-restricted diet (Figure 2B). The concentration of four amino acids including two NEAAs (glycine and alanine) and two EAAs (methionine and isoleucine) significantly increased in aphids fed the amino acid-restricted diet (Figure 2B). Notably, the concentrations of aspartate had the largest fold change in aphids fed the Half diet compared with the Control diet (eightfold decrease) (Figure 2B). Four types of sugar were detected in aphids fed both Half and Control diets, including trehalose, glucose, sucrose, and fructose (Figure 3A). Interestingly, we found that trehalose, glucose, and sucrose all accumulated at higher concentrations in aphids fed the Half diet compared with the Control diet (Figure 3A). There was no significant difference in total protein content between aphids fed the Half and Control diets (Figure 3B).

### Illumina Sequencing and Read Assembly

The Illumina sequencing of aphids fed the Half and Control diets produced an average of 63,730,408 and 64,779,453 raw reads, respectively. After cleaning and quality control, an average of 62,349,773 and 63,580,526 clean reads was obtained, respectively. The summary of sequence analyses of aphids fed the Half and Control diets is shown in Table 1. All reads were deposited in the NCBI Short Read Archive (SRA, the accession number SRP241482). Sequences from the two libraries were combined and 17,453 genes were finally obtained with a mean length of 1953 nucleotides (nt). The length range of genes was 23 to 49,286 nt. The N50 of genes is 2492 nt.

**TABLE 1:** Summary of transcriptome parameters of *Myzus persicae* fed the Half or Control diet.

	Half diet			Control diet		
		
	1^a^	2	3	1	2	3
Number of raw reads	70242158	53281054	67668012	61780850	73730246	58827262
Number of clean reads	68825240	51916626	66307454	60658622	72518450	57564506
Q20 percentage (%)	97.78	97.9	97.85	97.88	97.65	97.79
Q30 percentage (%)	93.72	94.08	93.92	93.97	93.44	93.81
GC content (%)	40.34	41	40.75	40.58	39.39	40.94
Number of genes	13912	13625	13877	14035	14169	13986
Mean length of genes (nt)	2191	2209	2192	2183	2173	2182

*^a^Values combined all independent biological replicates.*

### Functional Annotation of DEGs Between Aphids Fed the Half and Control Diets

**FIGURE 4:**
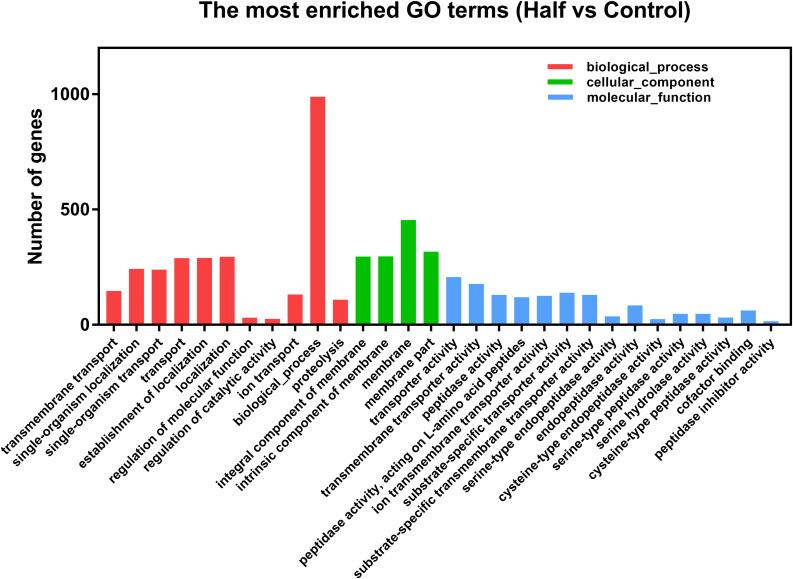
Top 30 gene ontology (GO) terms of DEGs of *Myzus persicae* fed the Half diet.

After differential expression analysis, we found 1460 genes with significantly different expression levels in *M. persicae* fed the Half diet, when compared with Control diet. Detailed information for all DEGs is shown in Supplementary Table S2. The DEGs between aphids fed the Half and Control diets were functional annotated with GO enrichment analysis. The top 30 most enriched GO terms were shown (Figure 4). The detailed information for Top 30 GO terms is shown in Supplementary Table S3. Many GO terms within the molecular function category were included in top 30 enriched GO terms, namely, transmembrane transport, peptidase activity (acting on L-amino acid peptides), and cysteine-type peptidase activity, as well as serine-type peptidase activity (Figure 4).

### Enriched Pathways of DEGs of Aphids Under Two Dietary Amino Acid Concentrations

**FIGURE 5:**
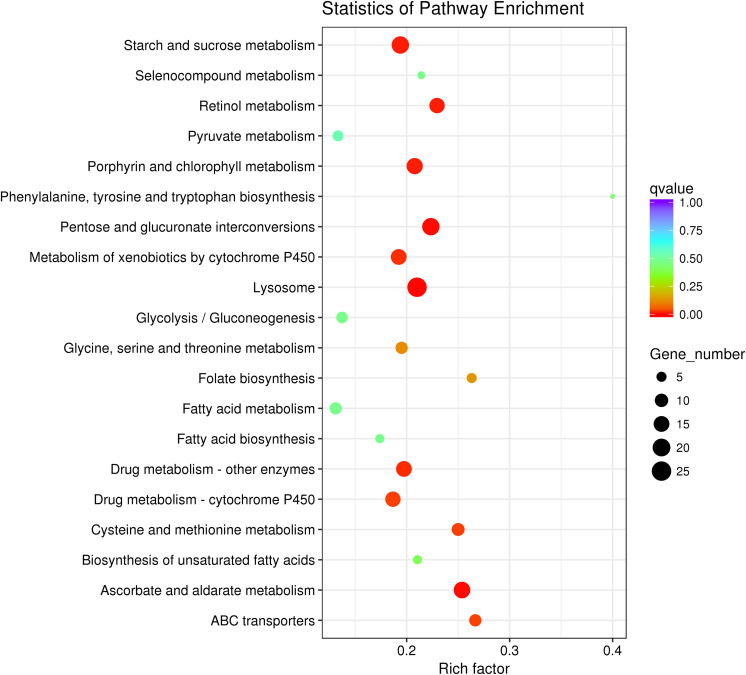
Top 20 enriched upregulated Kyoto Encyclopedia of Genes and Genomes (KEGG) pathways of *Myzus persicae* fed the Half diet.

After KEGG analysis, DEGs were mapped to 102 KEGG pathways and the top 20 upregulated KEGG pathways are shown (Figure 5). Detailed information regarding up- or downregulation of KEGG pathways is shown in Supplementary Table S4. The lysosome pathway containing 35 DEGs was the most enriched upregulated KEGG pathway (Figure 5). Notably, many DEGs were mapped to nutrient and energy metabolism pathways, including 10 DEGs related to cysteine and methionine metabolism. A further eight DEGs were related to glycine, serine, and threonine metabolism, and two were related to phenylalanine, tyrosine, and tryptophan biosynthesis pathways. Additionally, we observed enrichment in starch and sucrose metabolism and the glycolysis/gluconeogenesis pathways (Figure 5).

### Differentially Expressed of Genes Involved in Amino Acid, Sugar, and Protein Metabolism Under Two Dietary Amino Acid Levels

**FIGURE 6:**
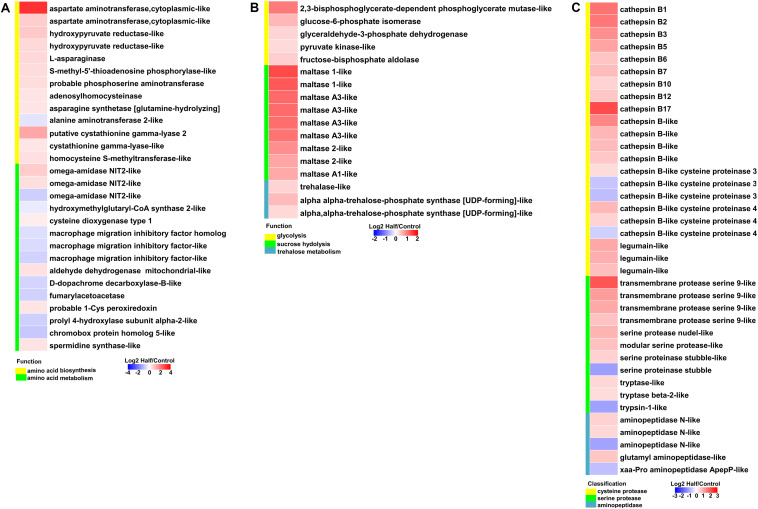
Heatmaps showing altered expression of genes involved in nutrient and energy metabolism in aphids fed the Half diet compared with Control diet. **(A)** Genes involved in amino acid biosynthesis and metabolism. **(B)** Genes involved in sugar metabolism. **(C)** Genes involved in protein degradation.

**FIGURE 7:**
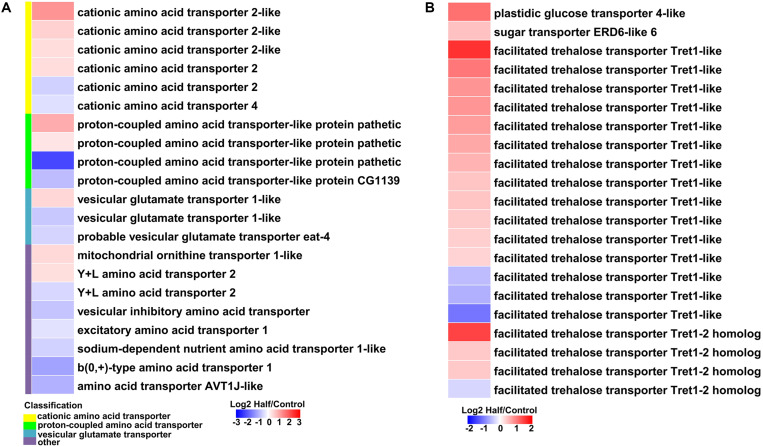
Heatmaps showing altered expression of genes involved in amino acid and sugar transport in aphids fed the Half diet compared with Control diet. **(A)** Genes involved in amino acid transport. **(B)** Genes involved in sugar transport.

With restricted access to dietary amino acids, aphids show extensive transcriptional reprogramming of genes involved in nutrient and energy metabolism. We observed upregulation of genes involved in amino acid biosynthesis, sugar metabolism, and transport, as well as protein metabolism in aphids fed the Half diet (Figures 6, 7).

Most genes involved in the biosynthesis of NEAAs (aspartate, asparagine, serine, and cysteine) were upregulated in aphids fed the Half diet (Figure 6A). Several upregulated genes related to biosynthesis of EAAs, such as methionine, included putative cystathionine gamma-lyase 2, cystathionine gamma-lyase-like, and homocysteine S-methyltransferase-like (Figure 6A). It is noteworthy that the gene encoding aspartate aminotransferase was the most highly upregulated (9.31-fold) in aphids fed the Half diet (Figure 6A). In addition, the expression of many amino acid transporter genes changed in aphids fed the Half diet, including the upregulation of nine and downregulation of 12 amino acid transporter genes (Figure 7A).

It is a remarkable fact that the transcript levels of sugar metabolism and transport genes were upregulated in aphids fed the Half diet, including facilitated trehalose transporter Tret1, maltase, glucose transporter, and many glycolysis genes (Figures 6B, 7B).

Furthermore, most cysteine protease genes including cathepsin B genes and legumain-like genes as well as serine protease genes were upregulated in aphids fed the Half diet (Figure 6C). Several aminopeptidase genes were also upregulated in aphids fed the Half diet (Figure 6C).

### Effects of Dietary Restriction of Amino Acids on the Expression Level of Genes Related to Hormone Signaling Pathway

To detect whether the expression of genes from hormone signaling pathways change in aphids fed an amino acid-restricted diet, we focused on the expression level of genes from JH, 20-hydroxyecdysone (20E), and insulin-like peptides (ILPs) signaling pathways. We found that dietary restriction of amino acid significantly affected the expression of several genes in these hormone signaling pathways (Table 2).

**TABLE 2:** Differential expression of genes from the hormone signaling pathway of *Myzus persicae* fed the Half diet.

Gene ID	Description	Log_2_foldchange	Adj. *P*-value
**JH signaling pathway**			
111039259	Krüppel homolog 1	–1.1799	1.11 × 10^–11^
**20E signaling pathway**			
111029901	Broad-complex core protein-like	0.6595	3.21 × 10^–8^
111031103	Ecdysone 20-monooxygenase-like	0.5043	9.58 × 10^–3^
111039298	Hormone receptor 4-like	–0.8330	4.55 × 10^–2^
111040386	Hormone receptor 4-like	–0.9412	3.54 × 10^–3^
**ILPs signaling pathway**			
111026202	Insulin-like peptide 5	–0.7334	6.39 × 10^–9^
111031688	Neural/ectodermal development factor IMP-L2	0.6551	5.45 × 10^–8^

### Validation of Transcriptome Data by RT-qPCR

**FIGURE 8:**
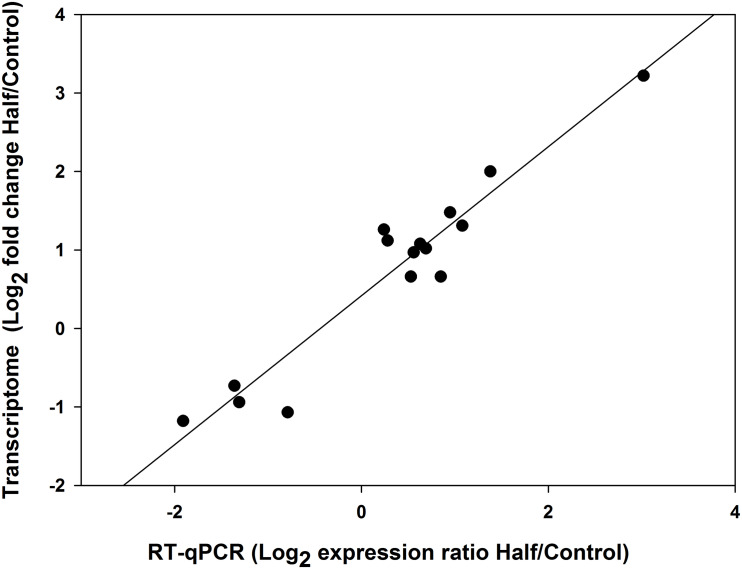
Pearson’s correlation of gene expression changes of *Myzus persicae* measured using RNA sequencing (RNA-seq) and real-time quantitative PCR (RT-qPCR).

All the 11 upregulated genes and four downregulated genes in *M. persicae* fed the Half diet selected for validation showed significant upregulation and downregulation in RT-qPCR, respectively, and gene expression results for both methods are listed in Table 3. The 15 selected genes showed a significant correlation (*r* = 0.959, *p* < 0.01, Spearman correlation coefficient) between RNA-seq data and RT-qPCR results (Figure 8), thus validating the analysis of RNA-seq in this study. Target gene-specific primers for RT-qPCR are shown in Supplementary Table S5.

**TABLE 3:** Transcriptome date validation by RT-qPCR.

Gene	Annotation	RT-qPCR data		Transcriptome data	
			
		Log_2_ expression ratio	Regulation	Log_2_ expression ratio	Regulation
		Half/Control		Half/Control	
111036240	Aspartate aminotransferase	3.02	Up	3.22	Up
111028794	Cationic amino acid transporter 2-like	0.24	Up	1.26	Up
111030241	Proton-coupled amino acid transporter-like protein pathetic	0.56	Up	0.97	Up
111032907	Cathepsin B5	0.63	Up	1.08	Up
111031294	Legumain-like	0.69	Up	1.02	Up
111028373	Transmembrane protease serine 9-like	1.38	Up	2.00	Up
111041315	Aminopeptidase N-like	−0.79	Down	−1.07	Down
111039505	Maltase 1-like	1.08	Up	1.31	Up
111042207	Facilitated trehalose transporter Tret1-2 homolog	0.95	Up	1.48	Up
111033224	Plastidic glucose transporter 4-like	0.28	Up	1.12	Up
111039259	Krüppel homolog 1	−1.91	Down	−1.18	Down
111029901	Broad-complex core protein-like	0.53	Up	0.66	Up
111040386	Hormone receptor 4-like	−1.31	Down	−0.94	Down
111026202	Insulin-like peptide 5	−1.36	Down	−0.73	Down
111031688	Neural/ectodermal development factor IMP-L2	0.85	Up	0.66	Up

## Discussion

To adapt to changing nutritional conditions, insects have evolved the ability to alter their growth and metabolism to coordinate them with nutrient availability. Low dietary amino acids availability usually causes reduced performance in aphids (Simpson et al., 1995; Ponder et al., 2000; Hunt et al., 2009). However, few studies have explored the molecular basis of these effects on aphid performance and physiology.

First, our results reveal that an amino acid-restricted diet has major impact on the performance of *M. persicae* including lower weight (Figure 1A) and declined fecundity (Figure 1B) which were also reported in *A. pisum* and *R. padi* (Simpson et al., 1995; Ponder et al., 2000). However, the *r*_*m*_ of *M. persicae* fed the *A. thaliana aap6* mutant (a mutant with reduced total amount of phloem sap amino acids to around 30% of wildtype plants) was only slightly reduced (Hunt et al., 2009). The differences in aphid performance between previous reports and our study may be explained by different feeding rates of aphids on plant and artificial diet. The feeding rate of *M. persicae* on mustard or radish seedlings was twice that of *M. persicae* on artificial diet (Mittler, 1970). In addition, aphids might utilize yet uncharacterized nutrients in plant phloem sap, thereby compensating for amino acid restriction. Second, we identified possible mechanisms that may be responsible for adaptation to the restriction of dietary amino acids in aphids.

Like most animals, aphids cannot synthesize EAAs, which are usually in short supply in phloem sap. In such cases, aphids require their symbionts *Buchnera* to synthesize EAAs from the NEAAs and sugar provided in the aphid diet (Febvay et al., 1999; Hansen and Moran, 2011). However, *Buchnera* has no transcriptional regulator of amino acid biosynthesis in its genome (Shigenobu et al., 2000), and further studies confirmed that *Buchnera* indeed lacks strong and specific transcriptional response to the manipulation of dietary amino acid supply for their aphid hosts (Moran et al., 2005; Wilson et al., 2006). These results led us to hypothesize that host aphids may have strong and specific transcriptional responses to dietary amino acid restriction. It is well understood that seven NEAAs (glutamate, aspartate, serine, glutamine, alanine, proline, and asparagine) are not synthesized by *Buchnera*; thus, the host aphid must synthesize these NEAAs and/or obtain them from diet (Hansen and Moran, 2011). Four of these NEAAs (glutamate, aspartate, serine, and glutamine) are particularly important as they are necessary nitrogen-containing substrates for the biosynthesis of EAAs by *Buchnera* (Hansen and Moran, 2011). Therefore, it is reasonable to speculate that upregulation of genes from amino acids biosynthetic pathways, particularly those involved in NEAAs biosynthesis (Figure 6A) is necessary for adjusting NEAAs profile to fit the needs of both itself and *Buchnera* under dietary amino acid restriction. Furthermore, amino acid transporters play an important role in facilitating the exchange of amino acids between aphid and *Buchnera* (Price et al., 2011, 2014). Differential expression of many amino acid transporter genes (Figure 7A) also indicates that aphids regulate amino acid biosynthesis under dietary amino acid restriction. Given they remain unaltered in total amount (Figure 2A), the amino acids present some changes in profile (Figure 2B), which further suggests that the decreased part of NEAAs may contribute to the synthesis of EAAs and/or other NEAAs.

Glucose can produce the precursors for the chemical constituents (e.g., nucleotides, amino acids, and lipids) by glycolysis, and these macromolecules are required for cell division (Lunt and Vander Heiden, 2011). Abundant glucose in bacteriocytes can fuel glycolysis in both aphid and *Buchnera*, resulting in abundant ATP and favoring amino acid biosynthesis, which has been proposed in study of aphid gene expression (Hansen and Moran, 2011). Therefore, the elevated expression of sugar metabolism genes (Figure 6B) and accumulation of sugars (particularly glucose) (Figure 3A) may maintain the levels of glycolytic intermediates necessary for supporting amino acid biosynthesis and provide enough ATP in aphids fed the Half diet. Given that phloem sap is rich in carbohydrates (Douglas, 2006), this upregulation of sugar metabolism could represent a survival strategy for aphids facing reduced dietary amino acids availability. Therefore, aphids can regulate the expression of genes related to amino acid biosynthesis, amino acid transport, and glycolysis under dietary amino acid restriction. The strong and specific transcriptional responses observed here are consistent with the perspective that host aphids can affect EAAs output by regulating the supply of precursor nitrogen (NEAAs) and carbon (glucose) to *Buchnera* spp. as proposed by Thomas et al. (2009) and support the results found for the pea aphid *A. pisum* (Price et al., 2014).

Cathepsin B, a kind of cysteine protease, and serine protease both could process exogenous polypeptides to form amino acids in several phloem-feeding hemipterans (Foissac et al., 2002; Cristofoletti et al., 2003; Rahbé et al., 2003; Deraison et al., 2004; Pyati et al., 2011). Furthermore, cysteine proteases are upregulated to minimize the effects of plant protease inhibitors (PIs) in some insects (Mosolov et al., 2001; Zhu-Salzman et al., 2003; Chougule et al., 2005). The cathepsin B and serine protease genes were upregulated in aphids fed the amino acid-restricted diet (Figure 6C). However, no PIs and exogenous polypeptides are present in the artificial diet used in our study. Therefore, we inferred that upregulation of these proteases may partially compensate for the shortage of dietary amino acids by enhancing the degradation of endogenous polypeptides into free amino acids in *M. persicae*.

In addition, the altered expression of genes from hormone signaling pathway may help aphids regulate growth and development to coordinate amino acid availability. The insect ILPs, as nutritional sensors, respond to dietary amino acid levels to adjust growth rate and reproduction (Wu and Brown, 2006; Smykal and Raikhel, 2015; Koyama and Mirth, 2018). For instance, *Drosophila* display a severe reduction in expression level of *Ilp5* when feeding on amino acid-restricted diet (Géminard et al., 2009). The downregulated *Ilp5* in *M. persicae* (Table 2) suggested a substantial response to restricted diets. Similarly, JH signaling positively affected by nutritional status in many insects (Stay, 2000; Noriega, 2004; Nouzova et al., 2011). The Krüppel homolog 1 (*Kr-h1*) is an insect anti-metamorphic factor and its gene expression is stimulated by JH (Minakuchi et al., 2009). Therefore, lower level of JH causing by amino acid restriction might be the possible reason leading to lower expression of *Kr-h1* in *M. persicae* fed the Half diet (Table 2). In contrast, 20E concentration is generally negatively affected by nutritional status in insects (Terashima et al., 2005; Johnson et al., 2014). The Shade (*Shd*) is an insect hydroxylase that transforms ecdysone into the active form 20E (Petryk et al., 2003). The broad-complex core protein (*Br-c*) is a downstream transcription factor of ecdysone signaling belonging to the 20E-induced regulatory gene (Gonzy et al., 2002; Piulachs et al., 2010). Therefore, higher expression levels of *Br-c* and *Shd* (Table 2) might be necessary for maintaining higher level of 20E in *M. persicae* under dietary amino acid restriction.

Taken together, our results revealed that the upregulation of genes involved in amino acid biosynthesis, glycolysis, and protein degradation, as well as the altered expression of genes related to hormone signaling pathways, may facilitate the adaptation of *M. persicae* to the restriction of dietary amino acids. Finally, our results regarding the effects of restriction of dietary amino acids on metabolic networks and hormone signaling pathways of *M. persicae* raise further research perspectives on (i) the response of metabolic networks to other nutritional stresses, (ii) the role of proteases in host adaption for both *M. persicae* and other aphid species, and (iii) the mechanism of hormone regulation of aphids in the adaption to different host plants. In addition, as pointed by Machado-Assefh et al. (2015), it should be considered that artificial diets affect the aphids’ feeding behavior and salivary gene expression because of the striking contrast of aphids feeding mode between artificial diet (feeding-in active mode) and phloem sap (feeding-in passive mode). Thus, a rigorous comparison of gene expression in aphids on artificial diet and host plant will be necessary for future investigations using artificial diet as a tool to appreciate the generality of the aphids’ transcriptomic response to various stresses.

## Data Availability Statement

The datasets generated for this study can be found in the SRP241482.

## Author Contributions

JW, T-XL, and H-HC conceived the idea and designed the research. JW, HL, and Z-FZ performed the experiments. JW and Z-FZ analyzed the data. JW wrote the manuscript. All authors revised and approved the manuscript.

## Conflict of Interest

The authors declare that the research was conducted in the absence of any commercial or financial relationships that could be construed as a potential conflict of interest.
