# Supplementary material for: Performance and Transcriptional Response of the Green Peach Aphid Myzus persicae to the Restriction of Dietary Amino Acids
Source: Front Physiol. 2020 May 25;11:487. doi: 10.3389/fphys.2020.00487 (PMC7261896; doi:10.3389/fphys.2020.00487)
Supplement: Supplementary file 1 [file Table_1.PDF]

Table S1. Composition of Half[a] and Control[b] Diet.

Components	Contents	Components	Contents
**Amino acids stock solution (mg/100 ml)**			
Alanine	178.70	Lysine mono HCl	350.96
Arginine	245.00	Methionine	72.37
Asparagine, H_2O	298.46	Phenylalanine	170.00
Aspartic acid	88.25	Proline	129.30
Cysteine	29.56	Serine	124.32
Glutamic acid	149.34	Threonine	127.08
Glutamine	445.61	Tryptophane	42.68
Glycine	166.58	Tyrosine	38.59
Histidine	100.70	Valine	190.84
Isoleucine	164.75	β-Ala	6.24
Leucine	231.53	Orn mono HCl	9.44
**Vitamins stock solution (mg/100 ml)**			
p-aminobenzoic acid	10.00	i-inositol	42.00
Biotin	0.10	Nicotinamide	10.00
D-calcium pantothenate	5.00	Pyridoxin HCl	2.50
Choline chloride	50.00	Riboflavin	0.50
Folic acid	1.00	Thiamine di-HCl	2.50
**Trace metals stock solution (mg/100 ml)**			
$CuSO_4\ 5H_2O$	0.47	Calcium citrate	10.00
$FeCl_3\ 6H_2O$	4.45	Cholesteryl benzoate	2.50
$MnCl_2\ 4H_2O$	0.65	KH_2PO_4	250.00
NaCl	2.54	$MgSO_4 \cdot 7H_2O$	121.00
$ZnCl_2$	0.83	L-Ascorbic acid	100.00
**Diet formula**			
**Half**		**Control**	
Amino acids stock solution	25.00 mL	Amino acids stock solution	50.00 mL
Vitamins stock solution	10.00 mL	Vitamins stock solution	10.00 mL
Trace metals stock solution	10.00 mL	Trace metals stock solution	10.00 mL
Sucrose	34.23 g	Sucrose	34.23 g
pH (adjusted with KOH)	7.4	pH (adjusted with KOH)	7.4
Final volume	100.00 mL	Final volume	100.00 mL

[a]Half was prepared with amino acids present as 50% of Control.

[b]Refer to Febvay *et al* (1988).
