# Supplementary material for: Performance and Transcriptional Response of the Green Peach Aphid Myzus persicae to the Restriction of Dietary Amino Acids
Source: Front Physiol. 2020 May 25;11:487. doi: 10.3389/fphys.2020.00487 (PMC7261896; doi:10.3389/fphys.2020.00487)
Supplement: Supplementary file 5 [file Table_5.pdf]

Table S5. Primer sequences for qRT-PCR.

Gene ID	Annotation	Primer	Sequence (5'→3')	Product length (bp)
111036240	aspartate aminotransferase	Forward (F)	CCTCCAAACCACGGTGCTAA	146
		Reverse (R)	GCACCTTCATCCTCGAGAGC	
111028794	cationic amino acid transporter 2-like	Forward (F)	ACACAGGCCCCCTATCTGAA	122
		Reverse (R)	CCGACGGCCACATAACTGTA	
111030241	proton-coupled amino acid transporter-like protein pathetic	Forward (F)	CGGCCTCCGAAAAACACAAG	128
		Reverse (R)	TACGACGCCCATCACTATGC	
111032907	cathepsin B5	Forward (F)	CGGTCTAGTAACTGGAGGAGAC	104
		Reverse (R)	CCAACGGTTGATCTGAGGAT	
111031294	legumain-like	Forward (F)	GCATCAGGACCCAGGGAAAA	121
		Reverse (R)	ATACCGTTGCGTCCCAGTTT	
111028373	transmembrane protease serine 9-like	Forward (F)	GTCAGTTGCACTGATTGCCG	132
		Reverse (R)	GACGGTGCGGCCTTTAAATC	
111041315	aminopeptidase N-like	Forward (F)	CCGAGAACAGTTTTGTTTTGTGG	150
		Reverse (R)	TCAAAATGAGCTGTGCCTACAC	
111039505	maltase 1-like	Forward (F)	AATAGTACTCCGGAAGGTTTTCAG	102
		Reverse (R)	TGGATGGTCGTGGTTTTGGA	
111042207	facilitated trehalose transporter Tret1-2 homolog	Forward (F)	TGATGCTGTTCGAGGGGTTC	136
		Reverse (R)	ATGTACGACGGTATGACGGC	
111033224	plastidic glucose transporter 4-like	Forward (F)	GCCGAGAATGAGTTGACACG	149
		Reverse (R)	GAGCTTTAGATGGCGACCGA	
111039259	Krüppel homolog 1	Forward (F)	GGAAAATTGCACCGGCACAT	129
		Reverse (R)	CGGTTTTTCGCCAGTGTGAG	
111029901	broad-complex core protein-like	Forward (F)	TACGTATACCAGGCGGGACA	133
		Reverse (R)	TCCTCGTCGTCCCTCAGATT	
111040386	hormone receptor 4-like	Forward (F)	CGGTTGTGTTTTAGAATTTGGACC	146
		Reverse (R)	GGTCACACGGGGTACTTGTT	
111026202	insulin-like peptide 5	Forward (F)	TGTGCAGCAGTCAGTCAGTT	129
		Reverse (R)	TCGCACCTGTAATTCGTGGG	
111031688	neural/ectodermal development factor IMP-L2	Forward (F)	GCAACGCCTTCAGACCCTAT	116
		Reverse (R)	TGGTTCAGTTGTACCCCACG	
111032347	60S ribosomal protein L7 (Reference gene)	Forward (F)	TGCCGGAGTCTGTACTCAAA	92
		Reverse (R)	CACGCGTTCTTTACGTTCCT	
